# Investigating the Interaction Profile of Unconjugated Ubiquitin: Chemical Biology and Affinity Enrichment Mass Spectrometric Approaches

**DOI:** 10.1002/cbic.202500444

**Published:** 2025-08-29

**Authors:** Simon Maria Kienle, Katrin Stuber

**Affiliations:** ^1^ Departments of Biology and Chemistry Konstanz Research School Chemical Biology University of Konstanz Universitätsstr.10 78457 Konstanz Germany; ^2^ Translational Omics and Islet Biology Steno Diabetes Center Copenhagen Borgmester Ib Juuls Vej 83 2730 Herlev Denmark; ^3^ Faculty of Health and Medical Sciences Department of Cellular and Molecular Medicine Novo Nordisk Foundation Center for Protein Research University of Copenhagen Blegdamsvej 3B 2200 Copenhagen Denmark

**Keywords:** protein modifications, protein–protein interactions, proteomics, synthetic biology, ubiquitin

## Abstract

The covalent attachment of ubiquitin (Ub) to target proteins (ubiquitylation) represents one of the most versatile post‐translational modifications (PTM) in eukaryotic cells. Substrate modifications range from a single Ub moiety being attached to a target protein to complex Ub chains that can also contain Ubls (Ub‐like proteins) or chemical modifications like acetylation or phosphorylation. The entirety of this complex system is entitled as “the Ub code”. To regulate the Ub code, cells have an arsenal of enzymes to install, translate, and reverse these modifications. However, deciphering the Ub code is challenging due to the difficulty of generating defined Ub/Ubl−protein conjugates. In this mini review, an overview of chemical biology techniques for the generation of defined Ub variants and their subsequent application in affinity enrichment experiments to identify interacting proteins by mass spectrometry is provided. The main focus is on unconjugated Ub variants since they are not well understood even though a “second messenger”‐like function of those have been found. Finally, the opportunities to expand this approach to Ubl proteins are briefly discussed.

## Graphical abstract

This mini review provides insights into the synergistic power of chemical biology techniques for generating defined ubiquitin (Ub) variants and the use of affinity enrichment mass spectrometry to decipher the Ub code. It focuses on unconjugated Ub variants, which have emerged as additional signaling molecules in the Ub lexicon. Furthermore, it outlines the use of this approach with Ub‐like proteins. (Figure created in BioRender. (2025) https://BioRender.com/7vby989).

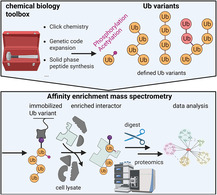

## 1 Introduction

**Figure 1:**
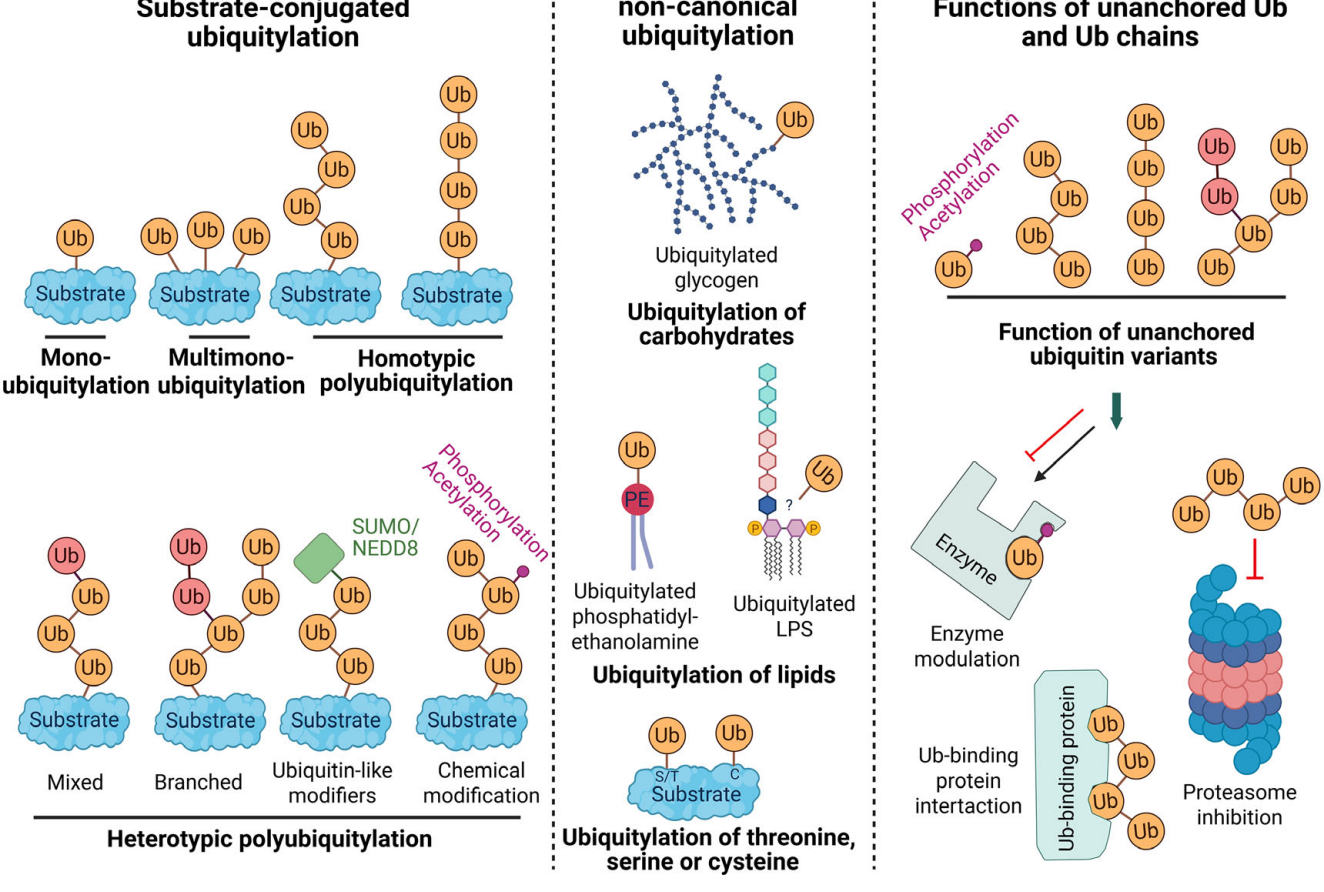
The diversity of ubiquitylation is reflected in the existence of canonical and non‐canonical Ub variants, as well as in unanchored Ub variants. Canonical ubiquitylation includes mono‐ and multimono‐ubiquitylation, as well as poly‐ubiquitylation—the latter can vary in its linkage types (red and orange Ub) and may incorporate mixtures of Ub/Ubl proteins (e.g., NEDD8 or SUMO) or chemically modified Ub (e.g., phosphorylated or acetylated). Non‐canonical ubiquitylation refers either to the attachment of Ub to non‐proteinogenic substances, such as sugars, lipids, like, for example, lipopolysaccharide (LPS), or to the conjugation of Ub to residues other than lysine and the initial methionine. In non‐amine ubiquitylation, Ub is, for example, attached to the side chains of threonine (T), serine (S), or cysteine (C) residues. Unanchored Ub variants are Ub molecules that are not attached to any specific substrate, and they can be as diverse as their anchored counterparts. These molecules have been found to modulate enzyme activity, bind to Ub‐binding proteins, and exert an inhibitory effect on the proteasome. (Created in BioRender. (2025) https://BioRender.com/3ruvnpo).

The covalent attachment of Ub to substrate proteins, by enzymes of the Ub cascade, is essential for nearly all pathways in a eukaryotic cell.^[^ ^1^ ^]^ The number and topology of Ub molecules attached to a substrate determine the specific fate of the ubiquitylated protein.^[^ ^2^ ^]^ Ub can also serve as its own substrate allowing to generate Ub chains. For this purpose, any of its seven lysine residues (K6, K11, K27, K29, K33, K48, K63) or the *α*‐amino group of the N‐terminal methionine (M1) can be used.^[^ ^3^ ^]^ This makes ubiquitylation one of the most versatile PTMs. The variety of different modifications is also referred to as “the Ub code” (**Figure** **1**).^[^ ^1b^ ^,^ ^2a^ ^]^ However, the discovery that Ub is also subjected to PTMs,^[^ ^2a^ ^,^ ^2c^ ^]^ like acetylation^[^ ^4^ ^]^ or phosphorylation,^[^ ^5^ ^]^ as well as the identification of non‐canonical ubiquitylation,^[^ ^6^ ^]^ where Ub is, for example, attached to hydroxyl‐groups of proteins, lipids,^[^ ^7^ ^]^ and sugars,^[^ ^8^ ^]^ added an additional layer of complexity to the Ub code. This complexity is further increased because unconjugated Ub can also regulate cellular pathways, by for example, modulating enzyme activity.^[^ ^9^ ^]^ In a simplified way, these unconjugated Ub variants can be viewed as “second messenger”‐like molecules (Figure 1).

The ability of cells to interpret the Ub code and to induce the respective responses relies mainly on ubiquitin‐binding proteins and their ubiquitin‐binding domains (UBDs).^[^ ^10^ ^]^ It is well‐known that certain UBDs recognize specific Ub variants, such as Ub chains with particular linkages.^[^ ^10a^ ^,^ ^11^ ^]^ However, for recently discovered and difficult‐to‐generate Ub variants, the specific binding proteins remain mainly unknown. Moreover, many interactions of ubiquitin‐binding proteins to Ub variants were studied *in vitro* in a case‐to‐case manner by using recombinant (truncated) interacting proteins.^[^ ^12^ ^]^ This is partly due to the challenge of generating stable and homogeneously modified Ub variants *in vivo*, which would enable the investigation of Ub variant‐selective interactions within a cellular system.^[^ ^12^ ^,^ ^13a^ ^]^ However, recent advances in chemical biology enable to generate many of those variants.^[^ ^13^ ^,^ ^14^ ^]^ These can be used to enrich interactors from crude cell lysates at near‐physiological conditions, allowing subsequently the identification of these interactors by mass spectrometry (MS).^[^ ^4a^ ^,^ ^12^ ^,^ ^15^ ^]^ This enables the proteome‐wide identification of Ub variant‐selective interactions and helps to associate specific cellular pathways with their respective Ub variants, which is particularly important for those that are less well‐characterized.^[^ ^4a^ ^,^ ^12^ ^,^ ^15^ ^]^ In contrast to studies about ubiquitylation of substrates and their respective Ub architectures, only a few studies focused on unconjugated or free Ub types and their functions (Figure 1). However, these free Ub types are now emerging as important cellular players, influencing, for example, enzyme activities through protein–protein interactions,^[^ ^9a^ ^,^ ^15b^ ^]^ highlighting the need for more detailed studies in this respect. Consequently, we focus here on the generation of free Ub variants, by chemical biology techniques, and their subsequent interactome characterization via affinity enrichment mass spectrometry (AE‐MS).

## 2 Chemical Biology Tools to Generate Free Ub Variants for AE‐MS

The whole AE‐MS approach for determining the respective interactome of a certain Ub variant is based on two pillars, the possibility to generate defined Ub variants and the subsequent AE‐MS.^[^ ^16^ ^]^ Especially the generation of defined Ub variants represents a profound challenge, particularly for Ub modifications which are enzymatically difficult to generate in high purity.^[^ ^12^ ^,^ ^14a^ ^,^ ^16^ ^]^ As an attractive alternative to enzymatic approaches, numerous (site)‐specifically modified Ub variants can be produced by chemical biology tools due to recent advances in that field.^[^ ^13^ ^,^ ^14^ ^,^ ^16a^ ^,^ ^17^ ^]^ In other words, the availability of tools, like genetic code expansion (GCE), native or chemical ligation, are now facilitating the generation of many different Ub variants, which can be used as affinity matrix to identify Ub variant‐specific binding proteins from crude cell lysate.

**Figure 2:**
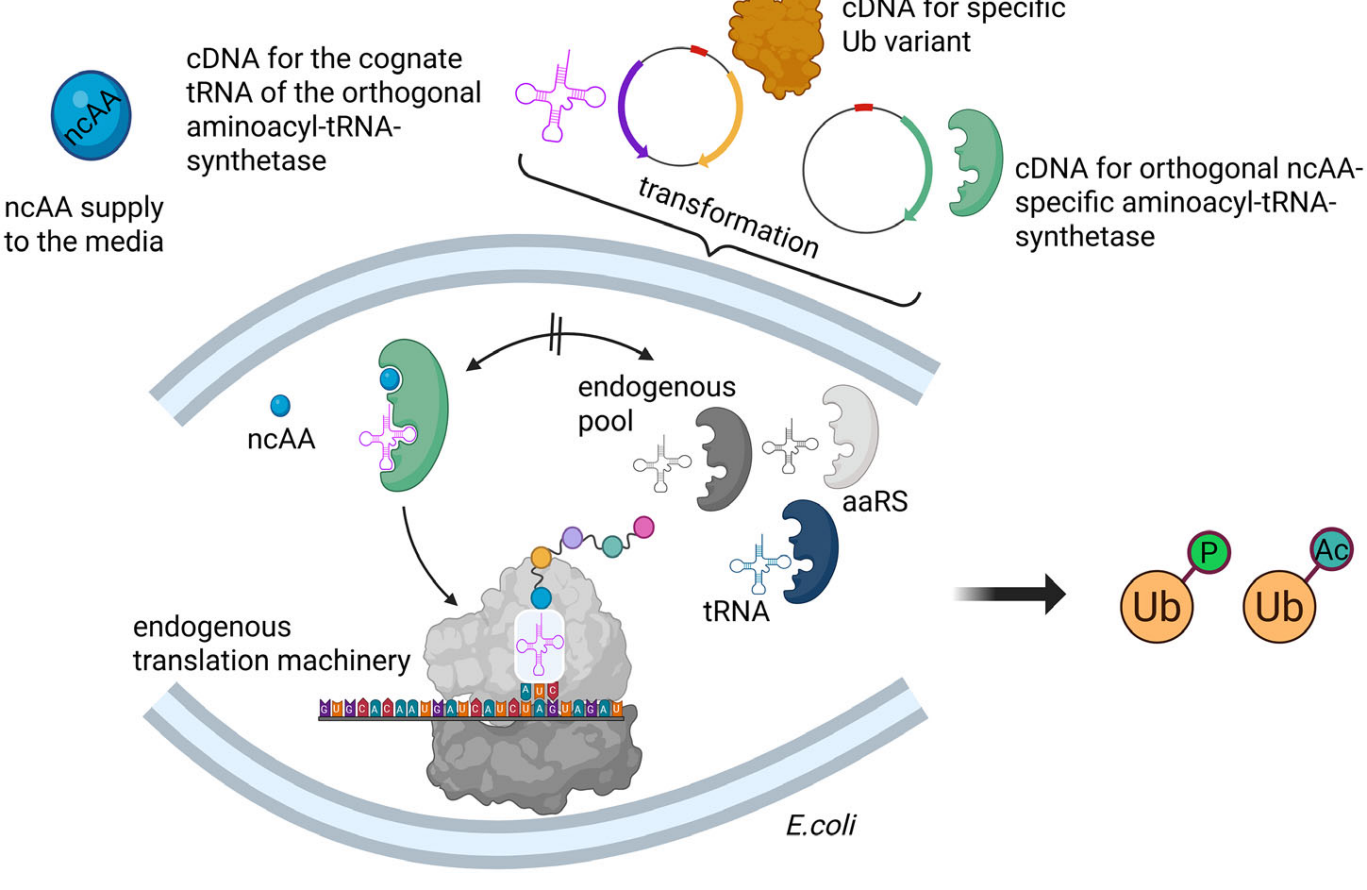
Genetic code expansion enables the generation of Ub variants that are modified in a homogeneous and site‐specific manner. It typically employs the host's endogenous translation machinery, allowing for the incorporation of a non‐canonical amino acid (ncAA) into Ub through an orthogonal aminoacyl‐tRNA synthetase (aaRS) that charges the ncAA onto its cognate tRNA. During mRNA translation, the ncAA is incorporated into a user‐defined site in a protein of interest in response to an amber stop codon. Importantly, the orthogonal aaRS does not aminoacylate or recognize any endogenous tRNAs in the host cell. (Created in BioRender. (2025) https://BioRender.com/j7al5sf).

GCE enables to artificially increase the chemical space of proteins, by incorporating non‐canonical amino acids (ncAA).^[^ ^18^ ^]^ This approach utilizes the translational machinery of a host organism to introduce an ncAA into a protein of interest in a site‐specific manner (**Figure** **2**) by reprograming the genetic code.^[^ ^18^ ^]^ In the context of AE‐MS, GCE is often used to site‐specifically incorporate PTMs or non‐hydrolzable analogs thereof into Ub by using the amber stop codon suppression method.^[^ ^4a^ ^,^ ^15b^ ^]^

Moreover, due to Ub's highly stable three‐dimensional structure, it tolerates harsh conditions allowing to generate many Ub variants by chemical synthesis, such as solid‐phase peptide synthesis (SPPS),^[^ ^13b^ ^,^ ^16^ ^]^ GOPAL (genetically encoded orthogonal protection and activated ligation),^[^ ^19^ ^]^ thiol chemistry^[^ ^13c^ ^,^ ^15c^ ^]^ or copper(I)‐catalyzed alkyne‐azide cycloaddition (CuAAC, aka ‘click chemistry’).^[^ ^13d^ ^,^ ^20^ ^]^ These approaches have not only been used to generate linkage‐defined Ub chains for the identification of Ub chain type/topology‐specific Ub binding proteins,^[^ ^12^ ^,^ ^15c^ ^c]^ but also to investigate the effect of Ub chain length on the respective interactome.^[^ ^12^ ^,^ ^15a^ ^]^ Thus, the combination of chemical biology and AE‐MS, to generate and to biologically characterize defined Ub variants, is a versatile and powerful tool.

The full capability of chemical biology in regard to Ub has been recently summarized in review articles.^[^ ^13a,f^, ^14^ ^]^

## 3 AE‐MS to Identify Interactors of Ub Polymers

**Figure 3:**
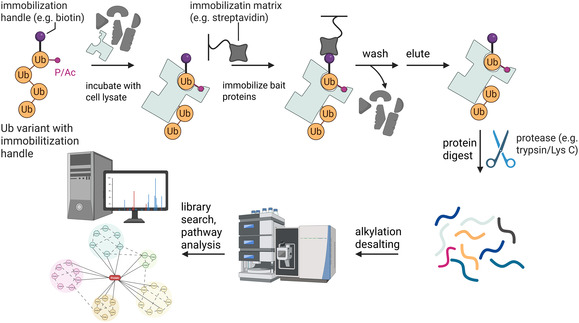
AE‐MS using ubiquitin variants follows a systematic workflow. First, the Ub variant equipped with an immobilization handle, such as biotin, is incubated with cell lysate to allow cellular interactors to bind to the Ub variant. After incubation, the Ub variant is captured on an immobilization matrix, for example, streptavidin‐coated beads, and unbound cellular proteins are removed through wash steps prior to eluting the Ub variant‐interactor complexes from the matrix. Next, these complexes are digested with endopeptidases, typically trypsin or trypsin/Lys‐C, as part of a bottom‐up proteomics approach. Notably, elution of the Ub‐interactor complexes prior to protein digestion is not always necessary, as on‐bead digestion can also be performed. Following digestion, the resulting peptides are reduced, alkylated, and desalted to prepare them for subsequent MS/MS analysis. Finally, peptide spectra are matched to a protein library to identify analyzed proteins, and significantly enriched interactors are subsequently used for pathway analysis and to construct interaction networks. (Created in BioRender. (2025) https://BioRender.com/bo4j4sb).

The approach to use chemical biology for AE‐MS with Ub has been pioneered by, *inter alia*, Ovaa and colleagues^[^ ^16b^ ^,^ ^21^ ^]^ and uses *in vitro* generated Ub variants as affinity matrix to enrich interacting proteins which are subsequently identified by high resolution MS/MS (**Figure** **3**).

For their approach Zhang et al. generated all possible linkage types of diubiquitin by click chemistry.^[^ ^12^ ^,^ ^16b^ ^]^ To do so, they applied linear Fmoc‐based SPPS, allowing them to incorporate an azide containing azido‐ornithine at the desired positions in the proximal Ub. For the distal Ub, they coupled, via the SPPS, an ethylene‐containing propargylamine to the C‐terminus of Ub. Subsequently, they used CuSO_4_ and sodium ascorbate, for *in situ* generation of Cu(I) ions, to perform the click reaction leading to diubiquitin linked via a triazole bond.^[^ ^12^ ^,^ ^16b^ ^]^ The triazole linkage comes with the benefit of being resistant against hydrolysis but simultaneously resembling the native isopeptide bond linkage.^[^ ^13d^ ^,^ ^22^ ^]^ This is important because (i) protein interactions are highly dependent on the structural integrity of the studied protein and (ii) the AE is performed in cell lysate containing deubiquitylases which would rapidly cleave the native isopeptide bond. The latter has been of particular importance in those studies since it allowed the identification of the deubiquitylase UCHL3 as a specific K27 interactor.^[^ ^12^ ^,^ ^21^ ^]^ Though, these features are not exclusive to triazole bonds but also to others, like isopeptide‐*N*‐ethylated bonds.^[^ ^13e,f^ ^]^

**Figure 4:**
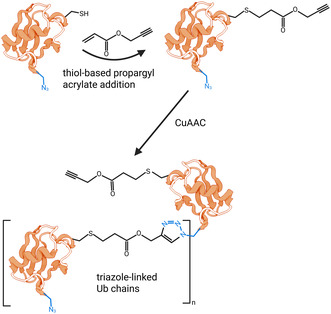
Site‐specific Ub chain generation through chemical biology approaches. An Ub molecule harboring an azide‐containing amino acid, introduced by, for example, SPPS or SPI, and a cysteine is incubated with propargyl acrylate, thereby enabling the site‐specific coupling of an alkyne moiety to Ub. Through Cu(I)‐catalyzed azide–alkyne cycloaddition (CuAAC), also known as click chemistry, ubiquitin chains of varying lengths can be generated. These chains are linked by a deubiquitylase‐resistant triazole linkage, rendering them suitable for in cell lysates use. (Created in BioRender. (2025) https://BioRender.com/7doavp1).

In contrast to the SPPS approach of Ovaa and colleagues, Scheffner et al. used thiol chemistry and selective pressure incorporation (SPI) to introduce the chemical moieties for click chemistry (**Figure** **4**). By doing so they were able to generate Ub chains connected via a triazole linkage at K27, K29 and K33.^[^ ^15a^ ^,^ ^15c^ ^]^ More specifically, in their approach the lysine of interest was replaced by a cysteine which can be functionalized through Michael addition with an ethylene‐containing propargyl acrylate. Of note here, wild‐type Ub does not harbor any cysteine, making this approach site‐specific. To functionalize Ub with an azide at its C‐terminus, Gly76 codon was deleted and Gly75 was replaced with a codon for methionine to enable the incorporation of azidohomoalanine (Aha) at every methionine position by SPI. Simultaneously, the fusion to a N‐terminal GST‐tag, which was removed after purification, enabled to delete the initial methionine in this Ub construct. Since both functional groups, azido and alkyne, have been incorporated in one Ub molecule, the click chemistry resulted in the efficient generation of Ub chains with different Ub polymer lengths.^[^ ^15a^ ^,^ ^15c^ ^]^ After affinity enrichment, Lutz et al. resolved the elution fractions by SDS‐PAGE and subsequently analyzed them by LC–MS/MS and label free quantification. This led to the identification of 70 interactors for K27 chains, while 44 and 37 interactors were identified for K29 or K33 chains, respectively.^[^ ^15c^ ^]^

In a subsequent study they further improved their approach and were able to separate the different Ub polymers according to their length. In this report, they showed that the length of a Ub chain generally has a major impact on Ub's ability to be selectively recognized by ubiquitin‐binding proteins.^[^ ^15a^ ^]^

Besides the use of authentic non‐hydrolyzable surrogates for certain Ub variants, another important point of concern is the choice of the immobilization handle and the attachment to the protein of interest. In most studies investigating Ub variants by AE‐MS, biotin and streptavidin‐coated beads were used as immobilization handle and stationary phase.^[^ ^12^ ^,^ ^15a^ ^,^ ^15c^ ^]^ Of note, introducing immobilization handles with *N*‐hydroxy‐succinimide (NHS) chemistry at primary amines, needs to be considered with caution since this additionally perturb the structural integrity of the protein of interest and thus influences the interactome. This is even more important when the studied protein is rather small, like Ub. The fact that introducing an immobilization handle via NHS can influence the interaction property of Ub, was also observed by Ovaa et al. in their study with diubiquitin.^[^ ^12^ ^]^ Hence, they introduced biotin, at the N‐terminus of Ub, with a small polyethylene glycol (PEG) spacer via SPPS to minimize the perturbation.

After enrichment, interactors and Ub bait molecules are either eluted or proteins are directly digested on beads by proteases, most commonly with the endopeptidases trypsin and Lys C. While the direct protein digest on beads represents a rather straightforward procedure, the elution of proteins immobilized with biotin‐streptavidin is more difficult. This is due to the strong interaction of biotin to streptavidin which requires a high excess of free biotin for elution. This excess of biotin needs to be removed prior to sample preparation for LC‐MS/MS. This can be achieved, among others, by in‐gel digest.

**Figure 5:**
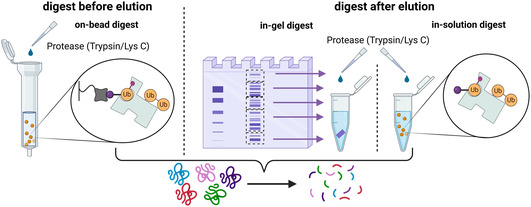
Protein digestion after affinity enrichment. After interactor enrichment, the proteins are digested for subsequent MS/MS analysis. The digestion can be performed either before eluting the Ub‐interactor complexes or after their elution. For the on‐bead approach, proteins are digested while still attached to the immobilization matrix, the resulting peptides are eluted subsequently. In the in‐gel digest, the Ub‐interactor complexes are first eluted and separated using SDS‐PAGE. Protein bands are then excised, and the gel pieces are subjected to alternating shrinking and swelling steps with organic and aqueous solvents, respectively, to enable uptake of a solution containing the appropriate proteases. Following digestion, the peptides are released from the gel. For the in‐solution digest, the Ub‐interactor complexes are eluted, and the proteins are digested directly in an aqueous solution with the corresponding proteases. (Created in BioRender. (2025) https://BioRender.com/pnq1ljv).

Proteolytic digestion can be achieved using three principal procedures: in‐gel, in‐solution, and on‐bead (**Figure** **5**). In both methods, in‐gel or in‐solution, the digestion is performed after eluting the Ub‐interactor complexes, whereas in the on‐bead approach, proteins are digested while still bound to the immobilization matrix. After the on‐bead digestion, the peptides are eluted from the matrix. Ovaa et al. performed a side‐by‐side comparison of on‐bead and in‐gel digest workflows with linear Ub chains.^[^ ^12^ ^]^ Here, they found a deeper sample coverage (twice as much identified proteins) with in‐gel digest in comparison to on‐bead. However, both workflows share the most prominent interactors, which led them to the decision to use an on‐bead digest approach since it is less laborious and more straightforward.

## 4 AE‐MS for Characterizing the Interactome of Post‐Translationally Modified Ub Variants

Besides Ub polymers, small PTMs of Ub, such as phosphorylation or acetylation, have been investigated in recent years.^[^ ^4a^ ^,^ ^15b^ ^]^ Especially Ub phosphorylation at serine 65 (Ub pS65) received attention since it plays an important role in the autophagy of damaged mitochondria, also known as mitophagy.^[^ ^5b^ ^,^ ^5c^ ^,^ ^23^ ^]^ Here, Ub pS65 allosterically activates the RING in‐between RING E3 ligase parkin, resulting in parkin‐mediated mitophagy.^[^ ^24^ ^]^ This fact highlights the second messenger‐like function of unconjugated Ub variants.^[^ ^15b^ ^]^

In a recent study, we were able to identify the interactome of Ub pS65, by using both a GCE approach as well as AE‐MS.^[^ ^15b^ ^]^ By GCE, we were able to incorporate both a hydrolyzable and a non‐hydrolyzable phospho‐serine ncAA, overcoming the disadvantage of using phosphatase inhibitors or amino acid substitutions to mimic phosphorylation. This is of special interest in case of Ub pS65 since the negative charge introduced by the phosphate results in pronounced structural rearrangement of Ub's C‐terminus, which is characteristic for Ub pS65.^[^ ^5a^ ^,^ ^24b^ ^]^ However, the frequently used phospho‐mimetics, glutamic acid or aspartic acid, are unable to resemble this.^[^ ^5a^ ^,^ ^15b^ ^]^ We showed that the non‐hydrolyzable phospho‐serine analog is a proper surrogate since it strongly mirrors the effect of phospho‐serine.^[^ ^15b^ ^]^ Consequently, a very similar interactomes of Ub with either the hydrolyzable or the non‐hydrolyzable phospho‐serine were found.^[^ ^15b^ ^]^

As an attractive alternative to the attachment of the immobilization handle via chemical tools, we equipped respective Ub variants with a N‐terminal Strep‐tag II for immobilization on Strep‐Tactin beads to perform AE‐MS. This alternative represents an easy and rather small alternative when no chemical tools can be applied.^[^ ^15b^ ^]^

Also, Ub acetylation has been recently investigated by GCE and AE‐MS.^[^ ^4a^ ^]^ Although Ub acetylation has been described earlier, little was known about its enzymatic regulation and its physiological consequences besides the inhibitory effect on Ub chain elongation.^[^ ^4^ ^,^ ^25^ ^]^ Like phosphorylation, studies investigating acetylation frequently used amino acid substitutions, mainly glutamate or arginine.^[^ ^26^ ^]^ However, strong evidence has recently emerged indicating that those substitutions are unable to fully mimic acetylation.^[^ ^4a^ ^,^ ^27^ ^]^ Thus, the generation of acetylated Ub variants by GCE represents a way to produce authentic surrogates, especially since acetyltransferases targeting Ub were previously unknown.^[^ ^4a^ ^]^ In the subsequent AE‐MS experiment, certain acetylated Ub variants were linked to particular cellular pathways, like NDP52‐mediated xenophagy.^[^ ^4a^ ^]^ The discovery of HDAC6 as a deacetylase for acetylated Ub, highlights the potential of AE‐MS to shed some light on the enzymology of less well‐characterized Ub modifications.

## 5 Expansion of AE‐MS Approaches to Ub‐Like Proteins

In line with Ub, also ubiquitin‐like proteins (Ubl), such as SUMO and NEDD8 are described to be modified by PTMs.^[^ ^28^ ^]^ However, little is known about their physiological function, especially when it comes to unanchored/free variants. This is in part due to difficulties generating specifically modified Ubls. One reason can be found in the lower stability of Ubls in comparison to Ub. This is especially true for NEDD8 even though NEDD8 represents, with 80% sequence similarity to Ub, Ub's closest relative.^[^ ^29^ ^]^ Despite that, some studies have been able to circumvent those hurdles. In our study, we focused on phosphorylated NEDD8 (pNEDD8) and showed by an AE‐MS approach that pNEDD8 binds the HSP70 complex more strongly than the unmodified counterpart.^[^ ^15b^ ^]^ In subsequent experiments, pNEDD8 was found to activate the HSP70 ATPase activity. These findings allowed to link NEDD8 phosphorylation to stress response signaling and to propose a “second messenger”‐like function of it.^[^ ^15b^ ^]^

## 6 Summary and Outlook

This mini review highlights the potential of combining chemical biology tools with AE‐MS for studying interacting proteins of distinct Ub/Ubl variants with the aim to decipher cellular pathways and involved enzymes. The advance of chemical biology methods now empowers the generation of many Ub modifications at will, enabling their detailed characterization. This is of high significance, since Ub and modified variants of it act as signaling molecules in many critical cellular pathways and thus it is crucial to determine the respective interactomes. Since Ub signal translation relies mainly on ubiquitin‐binding proteins, the combination of chemical biology with AE‐MS is a powerful tool to better understand the underlying mechanisms. In contrast to a case‐to‐case approach, this approach provides a holistic view of the cellular proteins, which interact with a certain Ub molecule.

**Figure 6:**
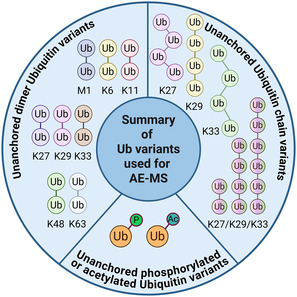
Several unanchored Ub variants, which are not attached to any specific substrate, have been employed in AE‐MS approaches to identify their interactomes. The interactomes of diubiquitin linked via a triazole bond at the initial methionine (M1) or at specific lysine (K) positions, as well as those of acetylated (Ac) or phosphorylated (P) Ub, have been investigated using AE‐MS. In addition, Ub chains of different lengths assembled through linkages at positions 27, 29, or 33 as well as Ub chains with defined lengths have been studied. (Created in BioRender. (2025) https://BioRender.com/zweu13u).

In future it will be interesting to see the application of chemical biology and AE‐MS beyond the studied Ub variants (**Figure** **6**) especially for Ub variants as, for example, Ub succiniylation and Ub conjugated to non‐proteinogenic substrates.^[^ ^6b^ ^,^ ^8^ ^,^ ^30^ ^]^ A crucial milestone for this is the availability of chemical tools to generate these Ub variants. Therefore, synthesis strategies, such as the use of sortase, are of key importance.^[^ ^31^ ^]^ For these Ub variants, the approach of chemical biology in combination AE‐MS will be very powerful in providing important insights into how these Ub variants are regulated as well as which proteins interact with them. Moreover, (photo)‐crosslinking procedures to capture weak or transient interactions which do not necessarily match binding affinities needed to be efficiently detected by standard AE‐MS approaches, can be applied to increase the amount of identified interactors. Additionally, these crosslinking techniques can even help to narrow down recognition elements in each interactor and thus provide information about indirect versus direct interactions.^[^ ^32^ ^]^

## Conflict of Interest

The authors declare no conflict of interest.
